# Mutation screening of *ASMT*, the last enzyme of the melatonin pathway, in a large sample of patients with Intellectual Disability

**DOI:** 10.1186/1471-2350-12-17

**Published:** 2011-01-20

**Authors:** Cecile Pagan, Hany Goubran Botros, Karine Poirier, Anne Dumaine, Stéphane Jamain, Sarah Moreno, Arjan de Brouwer, Hilde Van Esch, Richard Delorme, Jean-Marie Launay, Andreas Tzschach, Vera Kalscheuer, Didier Lacombe, Sylvain Briault, Frédéric Laumonnier, Martine Raynaud, Bregje W van Bon, Marjolein H Willemsen, Marion Leboyer, Jamel Chelly, Thomas Bourgeron

**Affiliations:** 1 Human Genetics and Cognitive Functions, Institut Pasteur, Paris, France; 2 CNRS URA 2182 "Genes, synapses et cognition", Institut Pasteur, Paris, France; 3 University Paris Descartes, Paris, France; 4 Institut Cochin, CNRS ULD 8104, Paris, France; 5 Inserm U 955, IMRB, Psychiatry Genetics, Creteil, F-94000, France; 6 Foundation Fondamental, Creteil, France; 7 Department of Human Genetics, Nijmegen Centre for Molecular Life Sciences, Radboud University Nijmegen Medical Centre, Nijmegen, The Netherlands; 8 Center for Human Genetics, University Hospitals Leuven, B-3000 Leuven, Belgium; 9 Service de Biochimie, INSERM U942, Hopital Lariboisière, Assistance Publique-Hopitaux de Paris, Paris, France; 10 Max Planck Institute for Molecular Genetics, Department Ropers, Berlin, Germany; 11 Service de Génétique Médicale, Hôpital Pellegrin, CHU de Bordeaux, Bordeaux, France; 12 UMR 6218, CNRS, IEM, équipe « génétique expérimentale et moléculaire », université d'Orléans, Orléans, France; 13 Centre hospitalier régional d'Orléans, Orléans, France; 14 Inserm U930 "Imaging and Brain", Tours, France; 15 University François-Rabelais, Tours, France; 16 CNRS ERL3106, Tours France; 17 Centre Hospitalier Régional Universitaire, Service de Génétique, Tours, France; 18 University Paris-East, Faculty of Medicine, UMR-S 955, Creteil, F-94000, France; 19 AP-HP, Henri Mondor-Albert Chenevier Group, Department of Psychiatry, Creteil, F-94000, France; 20 University Denis Diderot Paris 7, Paris, France

## Abstract

**Background:**

Intellectual disability (ID) is frequently associated with sleep disorders. Treatment with melatonin demonstrated efficacy, suggesting that, at least in a subgroup of patients, the endogenous melatonin level may not be sufficient to adequately set the sleep-wake cycles. Mutations in *ASMT *gene, coding the last enzyme of the melatonin pathway have been reported as a risk factor for autism spectrum disorders (ASD), which are often comorbid with ID. Thus the aim of the study was to ascertain the genetic variability of *ASMT *in a large cohort of patients with ID and controls.

**Methods:**

Here, we sequenced all exons of *ASMT *in a sample of 361 patients with ID and 440 controls. We then measured the ASMT activity in B lymphoblastoid cell lines (BLCL) of patients with ID carrying an ASMT variant and compared it to controls.

**Results:**

We could identify eleven variations modifying the protein sequence of *ASMT *(ID only: N13H, N17K, V171M, E288D; controls only: E61Q, D210G, K219R, P243L, C273S, R291Q; ID and controls: L298F) and two deleterious splice site mutations (IVS5+2T>C and IVS7+1G>T) only observed in patients with ID. We then ascertained ASMT activity in B lymphoblastoid cell lines from patients carrying the mutations and showed significantly lower enzyme activity in patients carrying mutations compared to controls (p = 0.004).

**Conclusions:**

We could identify patients with deleterious *ASMT *mutations as well as decreased ASMT activity. However, this study does not support *ASMT *as a causative gene for ID since we observed no significant enrichment in the frequency of *ASMT *variants in ID compared to controls. Nevertheless, given the impact of sleep difficulties in patients with ID, melatonin supplementation might be of great benefit for a subgroup of patients with low melatonin synthesis.

## Background

Intellectual disability (ID), is defined as IQ < 70 and is associated with functional deficits in adaptive behavior, such as daily-living skills, social skills and communication. It affects 1-3% of the general population and results from heterogeneous environmental, chromosomal and monogenic causes [1]. Besides the cognitive deficit, patients with ID often present with sleep disorders, which are persistent and a burden for the patients and their families. The most commonly reported disorders are delayed settling to sleep and frequent waking at night, with frequencies ranging from 58 up to 86% [2-4]. Several therapeutic strategies have been considered for treating sleep disturbances in ID. Among them, pharmacological use of melatonin was demonstrated to be efficient in several studies, recently reviewed in a meta-analysis [5]: exogenous melatonin appeared to decrease sleep latency and number of wakes per night, and increase total sleep time in individuals with ID. This positive effect of melatonin treatment could suggest that, in some patients with ID, endogenous melatonin level may not be sufficient to adequately set the sleep-wake cycles.

Melatonin is considered as a major biological signal of day-night rhythms, and thus a major endogenous "Zeitgeber" (time-giver). It is synthesized in the dark in the pineal gland from serotonin, first acetylated by aryl alkylamine N-acetyltransferase (AA-NAT) and then converted into melatonin by acetyl serotonin methyl transferase (ASMT also known as hydroxyindole O-methyltransferase or HIOMT). Besides sleep induction and circadian rhythms regulation, melatonin is also involved in various other physiologic functions, including immune response, antioxydative defense, metabolic regulations and memory [6-8]. Abnormal melatonin synthesis or signaling was reported as a risk factor for diverse medical conditions such as diabetes, circadian and psychiatric disorders [9-14]. Among these, autism spectrum disorders (ASD) - which are also often associated with ID and with sleep disorders - have been associated with low melatonin levels in at least four independent studies [15-18]. Melke *et al *[17] showed that melatonin deficit in patients with autism is correlated with low activity of the ASMT enzyme, and, in some patients, associated with mutations in the *ASMT *gene. This study provided the first insight into a molecular mechanism for melatonin deficit associated with neurodevelopmental disorders.

We hypothesized that patients with ID could carry deleterious *ASMT *mutations. If this was the case, these mutations might act as risk factors for sleep/circadian disorders and subsequently exacerbate the effect of independent genetic/environmental causes of ID. To address this question, we first screened the *ASMT *gene for rare variants in 361 patients with ID and 440 controls. For patients carrying *ASMT *mutations, we then measured the ASMT activity in B lymphoblastoid cell lines (BLCL) and if available provided information on sleep.

## Methods

### Subjects

In this study, we tested 361 clinically characterized male patients with established or putative X-linked ID, collected by the European XLMR Consortium (France, Belgium, Germany and the Netherlands). This panel included 182 established X-linked ID families characterized by at least two boys affected in two different generations and 113 brother-pair families with two or more affected brothers. Of 66 families, the exact number of affected males is not known, but linkage to the X chromosome was highly suspected. To study the frequency of *ASMT *mutations, we did not exclude from this cohort 68 previously described families with established X-linked mutations. The majority of the patients were from European ancestry. All samples were obtained after receiving informed consent. CGG expansions for fragile X syndrome, assessed by Southern blot analysis using DNA digested with EcoRI/EagI endonucleases and an StB12-3 probe corresponding to FRAXA, were excluded. Unrelated healthy controls of French origin (n = 220, 155 males, 65 females) were recruited among blood donors in two French university hospitals (Pitié-Salpêtrière and Henri-Mondor hospitals, Paris, France). Unrelated Swedish participants from the general population (n = 220, 142 males, 78 females) were recruited through advertisements. The local research ethics boards reviewed and approved the study. Informed consent was obtained from all participants.

### Screening of the *ASMT *gene for rare variations

DNA was extracted from blood cells by the phenol/chloroform method. All PCRs were performed with Qiagen HotStar Taq kit. Primers and PCR conditions have been described previously [17]. PCR products were sequenced with the BigDye Terminator Cycle Sequencing Kit (V3.1, Applied Biosystems) and then subjected to electrophoresis, using an ABI PRISM genetic analyzer (Applied Biosystems). For all non-synonymous mutations, genotyping was confirmed by sequencing of an independent PCR product. The nomenclature of genetic variations was determined according to reference protein sequence ENSP00000370627 in Ensembl database (345 aa). *In silico *functional predictions were assessed using PolyPhen (http://genetics.bwh.harvard.edu/pph/) and SIFT (http://sift.jcvi.org/) algorithms.

### Measurement of ASMT enzyme activity in BLCL

BLCL were established from EBV-transformed lymphocytes according to standard protocol, and grown at 37°C in RPMI-1640 medium (Life Technologies Inc.) supplemented with undialysed fetal calf serum, 2 mM glutamine, 2,5 mM sodium, 100 mg/mL streptomycin and 100 IU/mL penicillin, under standard conditions. ASMT enzyme activities were determined on BLCL pellets, at least in duplicate, by radioenzymology, as described previously [17], after lysis with 100 hemolytic units of a purified SH-activated toxin (streplolysin O, generously provided by Prof. J. Alouf, Institut Pasteur, Paris).

## Results

### Non-synonymous variants in the *ASMT *gene

We investigated whether rare non-synonymous variations in the *ASMT *gene could be identified in patients with ID by directly sequencing all *ASMT *exons in 361 patients with ID and 440 controls. Thirteen variants affecting exonic or splice-site sequences were identified, involving eight patients and eight controls (Table 1). Six of them were only found in patients and not in controls. Those include two splice site variants, which affect the splicing donor sites of intron 5 (IVS5+2T>C) and intron 7 (IVS7+1G>T). Both are predicted to introduce a stop codon shortly after the nucleotide change, and thus lead to a truncated protein. Interestingly, in five patients carrying an *ASMT *variant, a genetic cause for ID had been identified previously (Table 2). The patient with the N17K variant is a boy carrying a mutation in *ZNF41*. One of the two patients with an E288D variant is a boy diagnosed with FG syndrome. The index patient with the L298F variant is carrier of a mutation in the *MCT8 *gene. The patient with the IVS7+1G>T variant is a boy displaying the 24 bp duplication of *ARX *exon 2, and has one brother also carrying both *ASMT *variant and the same *ARX *duplication. The patient with the V171M variant is a boy carrying a duplication of *MECP2*. The other patients with *ASMT *variants have no known genetic anomaly involved in ID.

**Table 1:** *ASMT *mutations identified in 361 patients with ID and 440 controls.

Variant	ID Patients (n = 377)	Controls (n = 440)	Functional prediction (Polyphen/SIFT)
ID only			
N13H	1	0	Benign/tolerated
N17K*	1	0	Possibly damaging/tolerated
V171M	1	0	Possibly damaging/affects protein function
IVS5+2T > C	1	0	Damaging
IVS7+1G > T	1	0	Damaging
E288D	2	0	Benign/tolerated

ID and Controls			
L298F	1	2	Possibly damaging/affects protein function

Controls only			
E61Q	0	1	Benign/tolerated
D210G	0	1	Probably damaging/affects protein function
K219R	0	1	Benign/tolerated
P243L	0	1	Probably damaging/affects protein function
C273S	0	1	Probably damaging/affects protein function
R291Q	0	1	Probably damaging/tolerated

* N17K variant is rs17149149 and is mentioned in the SNP database at an allelic frequency of 6.7% in the Han Chinese population

**Table 2:** Clinical observations and ASMT activity in B lymphoblastoid cell line of patients with ID and *ASMT *mutations.

Individuals	Variants	ASMT activity (pmol/mg prot/30 min)	Clinical observations	Other known genetic anomalies
Patient D27	N13H	1.5	Mild ID. No other abnormalities or autistic features.	None
Patient P42	N17K	1.2	IQ:76, hyperkinesis, language delay, attention deficit and impulsivity, no epilepsy, no dysmorphic features.	ZNF41 mutation
Patient D33	V171M	1.6	Moderate ID, spasticity and severe language delay. No sleep-wake anomaly.	MECP2 duplication
Patient N6	IVS5+2T>C	0.9	Some autistic features; some compulsive behavior. Normal sleep pattern, although sleeps lightly and is easily wakened.	None
Patient P104	IVS7+1G>T	ND	Moderate ID, hyperactivity, attention deficit. No dysmorphic features. No evidence for sleeping problems or autistic features.	ARX duplication
Patient N79	E288D	0.9	Mild ID, epilepsy, dysmorphic features, scoliosis, strabismus, epilepsy, corpus callosum agenesis, subdural hygroma, hypermetry. Normal sleep pattern, although sleeps lightly. Some autistic features, with relatively low expressive communication and interpersonal relations, and compulsive behavior.	None
Patient T76	E288D	2.2	Severe ID, a few words. Dysmorphic features, hypotonia. Abnormal EEG, moderately enlarged lateral ventricles. No evidence for autistic features ('friendly behavior').	FG syndrome
Patient L45	L298F	1.7	Severe ID and no speech, however good social and eye contact. Never walked. No evidence for sleeping problems, or autistic features.	*MCT8 *mutation
Controls (n = 31) median (range)	WT	3.8 (0.2 - 9.5)		

Six variants were found only in controls and not in patients, and one variant (L298F) was found both in the ID group and in the control group. One variant, N17K (rs17149149), is mentioned in the SNP database at an allelic frequency of 6.7% in the Han Chinese population. When considering all the identified rare variations, we could not detect an enrichment of mutations in patients with ID compared to the control group (8/361 *vs *8/440 or 2.2% *vs *1.8%; p = 0.88; OR = 1.25 (0.46-3.46)).

### Impact of non-synonymous genetic variations on ASMT enzyme activity

**Figure 1:**
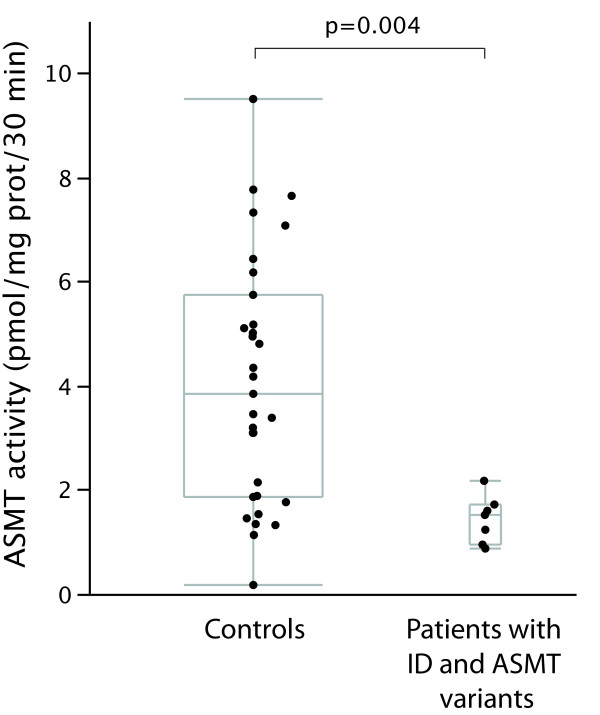
**ASMT activity in B lymphoblastoid cell lines of 31 unaffected controls and 7 patients with ID and *ASMT *mutation**. Grey boxes indicate medians and quartiles. Wilcoxon test: p = 0.004.

In order to investigate the functional effect of the genetic variations identified, ASMT enzyme activity was measured in BLCL of seven patients carrying *ASMT *variants (cells from the patient carrying variant IVS7+1G>T were not available) and 31 controls without coding mutations of *ASMT *(Figure 1). For six of the patients carrying variants, ASMT activity ranged in the first quartile of the control group (≤1.9 pmol/mg proteins/30 min). For the patient carrying variant E288D, ASMT activity ranged in the second quartile of the control group (2 - 3.3 pmol/mg proteins/30 min). Interestingly, for E288D, both PolyPhen and SIFT algorithms predicted little impact of the substitution on protein function. Surprisingly, the two unrelated patients carrying E288D variants displayed quite different BLCL ASMT activity (0.9 and 2.2 pmol/mg proteins/30 min), indicating that additional factors influence the enzyme activity.

On average, ASMT activity in BLCL of ID patients carrying variants was much lower as compared to control subjects without coding mutations of *ASMT *(1.4 ± 0.2 pmol/mg protein/30 min and 4.1 ± 0.4 pmol/mg protein/30 min, respectively, Wilcoxon test: p = 0.004). These data suggest that most variants identified in patients, although heterozygous, are associated with a low ASMT activity *ex vivo*.

## Discussion

Alterations of the melatonin pathway have been suggested as susceptibility factors to developmental disorders, and especially to ASD [15-17,19-23]. The mechanisms leading to ASMT/melatonin deficit in humans are most likely diverse, including genetic/epigenetic alterations. The impact of melatonin deficit on sleep and on the susceptibility to developmental disorders (such as ASD or ID) also remains unclear. It may involve its role as a circadian synchronizer and sleep inducer, its effects on synaptic plasticity, and/or its antioxidant properties [8,14,19,24,25]. Melatonin deficit may also alter and/or desynchronize many physiological processes, and indirectly exacerbate other pathological processes.

We could identify predicted deleterious variants in a subgroup of patients with ID, including two deleterious splice site variants of *ASMT *found only in patients with ID. The splice site mutation in intron 7 (IVS7+1G>T) was never observed before. The splice site mutation in intron 5 (IVS5+2T>C) was previously identified in patients with ASD and reported to be more frequent in patients compared to controls (6/749 vs 1/861; p = 0.04) [17,20,22]. In addition, biochemical studies indicated that several of the variants, although present at the heterozygous state, were associated with low ASMT activity and might thus impair melatonin synthesis *in vivo*. These results were consistent with the biochemical studies performed by Melke et al. on families carrier of the L298F and IVS5+2T>C mutations and presenting with a dramatic decrease in ASMT activity and blood melatonin concentration [17]. Nevertheless, despite these interesting findings, we could not detect *ASMT *mutation enrichment in patients with ID compared to the controls. Our results were similar to those previously reported in patients with ASD, for whom no significant enrichment in *ASMT *rare variants was found [17,20,22], although melatonin deficit is very frequently associated with this condition [15,16], and is correlated with low ASMT activity *in vivo *[17]. Low ASMT activities were also observed in BLCL of some controls subjects who did not carry a coding mutation of ASMT. Low ASMT activity can thus be observed even in the absence of coding mutations. For example, SNPs within the promoter were associated with low ASMT mRNA levels [17,21].

Several limitations exist in this study. First, the sample of patients with ID was initially collected for the identification of X-linked genes (e.g. families with multiple affected males). Therefore, this population is not representative of the broad diversity of patients with ID and is negatively biased for the identification of mutations in the *ASMT *gene, located on the pseudo-autosomal region 1 (PAR1) shared by the X and Y chromosomes. Another limitation is the sparse information that we could collect about sleep disorders. Further studies will be required to establish the precise link between *ASMT *variants, melatonin levels and sleep disorders.

## Conclusions

This study does not support *ASMT *as a causative gene for ID since we observed no significant enrichment in the frequency of *ASMT *variants in ID compared to controls. Nevertheless, we could identify patients with deleterious *ASMT *mutations as well as decreased ASMT activity. Given the importance of sleep difficulties in patients with ID, for this subgroup of patients, melatonin supplementation might be beneficial.

## Competing interests

The authors declare that they have no competing interests.

## Abbreviations

ASD: Autism Spectrum Disorders; ASMT: Acetyl-serotonin Methyl Transferase; BLCL: B Lymbhoblastoid cell lines; ID: Intellectual Disability; SNP: Single Nucleotide Polymorphism

## Authors' contributions

SM, CP, SJ participated in the sequencing the *ASMT *gene in patients and controls; CP, HGB, JML measured the ASMT activity; HVE, KP, SB, FL, MR, DL, AT, VK, AdB and JC were involved in the patient's recruitments and database managements. AD, SJ and ML were involved in the control's recruitments and database managements. TB, RD and JC conceived of the study, and participated in its design and coordination. CP, RD and TB drafted the manuscript. All authors contributed to and have approved the final manuscript.

## Pre-publication history

The pre-publication history for this paper can be accessed here:

http://www.biomedcentral.com/1471-2350/12/17/prepub
